# Associations between dentition status and cardiometabolic disease in adults: a cross-sectional analysis

**DOI:** 10.3389/froh.2026.1685373

**Published:** 2026-01-30

**Authors:** Rena Zelig, Matthew Swader, Riva Touger-Decker, Steven Singer, Hamed Samavat

**Affiliations:** Rutgers Health, Rutgers, The State University of New Jersey, Newark, NJ, United States

**Keywords:** cardiometabolic risk factors, cardiovascular diseases, dentition, diabetes mellitus, oral health, tooth loss

## Abstract

**Objectives:**

This study explored associations between dentition status and cardiometabolic diseases in adults.

**Materials and methods:**

Cross-sectional analysis was conducted using data from adults receiving routine care at an urban US Dental School clinic between January 1, 2020, and June 1, 2023. Associations between dentition status (measured by tooth loss and functional dentition [FD] status) and cardiometabolic diseases (diabetes mellitus [DM], hypertension [HTN], stroke, and cardiovascular disease [CVD]) were explored using binary logistic regression models while controlling for cardiometabolic risk factors [CMRFs].

**Results:**

The sample (*N* = 32,541) was 67.6% 40 years or older, 54.1% female, 51.7% White/Caucasian, 41.7% Black, 70.3% non-Hispanic/non-Latino, 69.6% overweight/obese; 16.0% reported tobacco use. The median number of remaining teeth was 26.0; 75.7% had FD. After adjusting for CMRFs, each additional tooth was associated with lower odds of DM (OR = 0.98, 95% CI = 0.98, 0.99), HTN (OR = 0.98, 95% CI = 0.98, 0.99), stroke (OR = 0.97, 95% CI = 0.96, 0.98), and CVD (OR = 0.97, 95% CI = 0.97,0.98). FD was associated with lower odds of DM (OR = 0.77, 95%CI = 0.70,0.84), HTN (OR = 0.78, 95% CI = 0.72, 0.850), stroke (OR = 0.65, 95% CI = 0.55,0.77), and CVD (OR = 0.71, 95% CI = 0.63–0.79). After adjusting for comorbid cardiometabolic conditions, having more teeth remained associated with lower odds of DM (OR = 0.99, 95% CI = 0.98, 0.99), HTN (OR = 0.99, 95% CI = 0.98, 1.00), and CVD (OR = 0.98, 95% CI = 0.97, 0.99). FD was associated with lower odds of DM (OR = 0.82, 95% CI = 0.68–0.92), HTN (OR = 0.84, 95% CI = 0.77, 0.92), and CVD (OR = 0.81, 95% CI = 0.71, 0.93).

**Conclusion:**

Tooth retention is associated with lower odds of having cardiometabolic disease. Enhanced knowledge and awareness of these associations can lead to improved cardiometabolic risk screening in interprofessional settings.

## Introduction

Cardiovascular disease (CVD) is the leading cause of death in the United States (US) and worldwide (1–3). While CVD-related mortality has decreased in recent years, 1 in 5 Americans still die from CVD, and globally, approximately 30% of deaths are attributable to CVD (1–3). Approximately 50% of adults in the US have a CVD-related diagnosis, including coronary heart disease, atherosclerosis, hyperlipidemia, hypertension (HTN), heart failure, arrhythmias, valvular diseases, and/or have experienced cardiovascular events such as myocardial infarction (MI) and stroke (1).

There are several independent modifiable and non-modifiable risk factors for the development of CVD (1, 4, 5). Non-modifiable risk factors include older age, male gender (although there is a higher incidence among women 80 years of age and older), being non-White (with the highest incidence among Black people), and being of Hispanic/Latino ethnicity (1, 4, 5). Modifiable risk factors include tobacco use, lack of physical activity (PA), overweight or obesity, and an unhealthy atherogenic diet (1, 4, 5). Cardiometabolic diseases, including HTN, diabetes mellitus (DM), and previous occurrence of an adverse cardiovascular event such as MI or stroke, also increase future cardiometabolic risk (CMR) and highlight the importance of secondary CVD prevention (4).

The association between tooth loss and CVD is multifaceted. Prior research has shown that dentition status impacts nutrient intake, which may further impact cardiometabolic health (6, 7). Cardiometabolic health and disease influences oral health through inflammatory pathways and conditions such as periodontal disease (PD) and metabolic syndrome (MS), resulting in tooth loss (8, 9). When the oral cavity is impaired by tooth loss, specifically the loss of a functional dentition (FD) (defined by the presence of 21 or more naturally occurring teeth) (10), biting and chewing are negatively affected (6, 7, 10–13). Impaired oral function leads to altered diet patterns and suboptimal diet quality, resulting in the avoidance or reduced intake of healthy foods rich in dietary fiber and nutrients, such as whole grains, fresh fruits, and vegetables. Concomitantly, there may be increased intake of easier-to-chew, calorically dense, ultra-processed foods, which are higher in saturated fats and added sugars (6, 7). The resulting diet pattern may be inconsistent with a heart-healthy dietary pattern recommended by the American Heart Association (14, 15).

Cardiovascular and systemic health may also influence oral health. Although the exact mechanisms for the underlying relationship between the conditions are unclear, inflammatory processes associated with CVD and PD can lead to tooth loss (16, 17). While PD is independently associated with CVD and atherosclerosis, no causation has been proven, and as such, it is unclear if CVD can worsen oral health or cause PD (18, 19). An extensive review of 22 systematic reviews and/or meta-analyses by Dietrich et al. exploring the associations between oral health and the risk of developing CVD found that the incidence of CVD is higher in individuals with PD than in those without PD, and that tooth loss is more prevalent among those with CVD (20). A meta-analysis conducted by Helfand et al. demonstrated a higher risk of CVD among those who were edentulous or with 10 or fewer teeth compared with those who had more than 10 teeth (HR: 1.34, 95% CI: 1.10–1.63) (21).

Multiple large cohort studies have explored associations between cardiometabolic risk factors (CMRFs) and tooth loss (22–26); however, only two have been conducted in US populations (25, 26), and the data were from surveys conducted over a decade ago. In these studies, dentition status was self-reported and not measured by clinical examination. Tooth loss was analyzed as a categorical variable with different categories, which increased the heterogeneity of the findings, and neither was specifically analyzed in terms of FD. Thus, this research aimed to explore the associations between tooth loss (clinically assessed as both the number of remaining teeth and the presence of a FD) and cardiometabolic diseases and their risk factors in U.S. adults treated in the current decade. We hypothesized that tooth retention would be associated with lower odds of cardiometabolic disease.

## Materials and methods

### Study population

This study was a cross-sectional analysis of anonymized patient data collected from adult patients aged 18–89 years who were treated at the Rutgers School of Dental Medicine (RSDM), an urban Northeastern US dental school clinic, between January 1, 2020, and June 1, 2023. The study sample comprised data from 32,541 patients with available data from their odontograms, enabling the calculation of their number of natural remaining teeth (NRT) and functional dentition status (FDS). All patient data for this research, including dentition status, sociodemographic, lifestyle, and clinical variables, were obtained from an electronic report from axiUm (EXAN, Vancouver, BC, Canada, available at https://www.exansoftware.com/axium/), the electronic health record (EHR) system used by the dental school. This study was approved by the institution's institutional review board (IRB) (Protocol #2021000687).

### Exposure assessment

The NRT was exported from the patient odontogram (which is completed by dentists or student doctors under dentist supervision) in AxiUm as a whole number ranging from 0 to 28 (excluding the third molars). It was also presented as a categorical variable, categorized into three groups: edentulous (no teeth), partial dentition (1–27 teeth), and complete dentition (28 teeth), for descriptive purposes (7). The presence of FD (defined as having ≥ 21 teeth) (10) was determined from the NRT and analyzed as a dichotomous variable (present or absent).

### Assessment of cardiometabolic risk factors (CMFRs)

Sociodemographic patient data included self-reported age, gender, race, and ethnicity, which are non-modifiable CMRFs. Age was categorized into four groups: 18–39 years old, 40–64 years old, 65–74 years old, and 75–89 years old (27–29). Gender was categorized as male or female. Race was categorized as White, Black or African American, Asian, or other (which included those who identified as Hawaiian/Pacific Islander, Native American/Alaska Native/Inuit, or more than one race). Ethnicity was categorized as Hispanic/Latino or Non-Hispanic/Non-Latino.

Lifestyle characteristics included modifiable CMRFs, including BMI and tobacco use. BMI was categorized according to the Centers for Disease Control and Prevention categories of underweight (BMI < 18.5 kg/m^2^), healthy weight (BMI 18.5 to <25 kg/m^2^), overweight (BMI 25 to <30 kg/m^2^), and obese (BMI ≥30 kg/m^2^) (30). Smoking status was categorized as yes/no.

### Outcomes assessment

The outcomes of interest included self-reported history of DM, HTN, Stroke, and CVD (inclusive of a history of MI), which were obtained from the patient's medical history in axiUm, and categorized as yes/no. The selection of these CMRFs and related clinical conditions (outcomes) was based on those that are collected as part of routine oral health care and available in the existing EHR, and which generally align with externally validated CMR assessment tools that have shown predictability of cardiometabolic outcomes, such as the Framingham Global Risk Assessment model and the Systematic Coronary Risk Evaluation (SCORE) model (27, 31–33). Other validated biochemical and anthropometric CMRFs, such as waist circumference, blood pressure (BP), and lipid measurements, which are included in other CMR assessment tools, were not available for this study and were therefore not included.

### Statistical analysis

All sociodemographic, lifestyle, and clinical characteristics (CMRFs and related diseases), as well as FD, were reported as categorical variables and presented using frequency distributions (n, %). Age, BMI, and the NRT were reported and analyzed as both categorical and continuous variables. As continuous variables, they were visually assessed for normality using histograms, box and whisker plots, and/or Q-Q plots. Since the data were not normally distributed, they were reported as the median and interquartile range (IQR). Where data were missing for lifestyle and clinical variables, they were excluded from that particular analysis. We did not impute missing data for race and ethnicity because these variables are sensitive, socially constructed, and not reliably predictable from other characteristics. Instead, we created a separate category for missing race and ethnicity and included it in all analyses.

As appropriate, bivariate analyses were conducted between dentition status and CMRFs using Mann–Whitney *U* and Kruskal–Wallis tests, adjusting for multiple testing through Bonferroni correction. Binary logistic regression models were constructed to examine the associations between the NRT and FDS with cardiometabolic diseases. NRT (on a continuous scale) and FDS (on a dichotomous scale, as FDS or lack of FDS) were considered predictor variables, and cardiometabolic diseases, including DM, HTN, stroke, and CVD, were considered outcome or dependent variables. All dependent variables included in the regression models were on a dichotomous scale (yes or no), with the absence of cardiometabolic disease considered the reference group. Three regression models were constructed; the first was the unadjusted/crude model, which accounted for multiple comparisons using the Bonferroni correction. The multivariable model 1, which was the model of interest, was adjusted for the modifiable and non-modifiable CMRFs (sociodemographic and lifestyle characteristics), including age, gender, race, ethnicity, BMI, and tobacco use. A third model (multivariable model 2) controlled for all confounders from multivariable model 1 (age, gender, race, ethnicity, BMI, and tobacco use), along with the other cardiometabolic diseases (DM, HTN, stroke, and CVD), where applicable, to address the issue of multimorbidity or coexistence of multiple health conditions. We also examined interactions between the potential confounders and NRT and FDS, and conducted adjusted stratified analysis if significant interactions were found. The strength of associations was reported as an odds ratio (OR) with corresponding 95% confidence intervals (CI).

An *a priori* power analysis was not conducted because the study utilized a convenience sample of previously collected data. All analyses were performed using SPSS version 31 (IBM Corp., Armonk, NY). *P*-value <0.05 was considered statistically significant. Where appropriate, *P* values were adjusted for multiple comparisons using the Bonferroni test.

## Results

Table 1 details the sample's sociodemographic, lifestyle, cardiometabolic, and dentition characteristics. The median NRT was 26.0 (IQR = 21.0, 28.0); 75.7% had a FD (10). Table 2 presents the associations between the NRT and the CMRFs and cardiometabolic diseases of interest, utilizing bivariate analyses. All associations were significant (*Ps* < 0.001), aside from gender (*P* = 0.523). Similarly, bivariate analyses revealed significant associations between the FDS and all CMRFs, as well as selected cardiometabolic diseases (*Ps* < 0.001), except for gender (*P* = 0.666) (Table 3).

**Table 1:** Sociodemographic, clinical characteristics and dentition Status of the sample.

Variable	*n* (%)
Age (*n* = 32,541)	
18–39 years old	10,539 (32.4)
40–64 years old	14,638 (45.0)
65–74 years old	4,835 (14.9)
75–89 years old	2,529 (7.8)
Gender (*n* = 32,541)	
Female	17,594 (54.1)
Male	14,947 (45.9)
Race (*n* = 32,541)	
White	7,148 (22.0)
Black or African American	5,760 (17.7)
Asian	697 (2.1)
Missing	18,713 (57.5)
Other^a^	223 (0.7)
Ethnicity (*n* = 32,541)	
Hispanic/Latino	4,230 (13.0)
Non-Hispanic/non-Latino	10,031 (30.8)
Missing	18,280 (56.2)
Body Mass Index (*n* = 24,147)	
Underweight (<18.5 kg/m^2^)	379 (1.6)
Healthy weight (18.5 to <25 kg/m^2^)	6,973 (28.9)
Overweight (25–29.99 kg/m^2^)	8,707 (36.1)
Obese (≥30 kg/m^2^)	8,088 (33.5)
Smoking Status (*n* = 23,617)	
No	19,839 (84.0)
Yes	3,778 (16.0)
History of Diabetes (*n* = 31,335)	
No	26,561 (84.8)
Yes	4,774 (15.2)
History of Hypertension (*n* = 30,636)	
No	20,871 (68.1)
Yes	9,765 (31.9)
History of Stroke (*n* = 31,282)	
No	30,075 (96.1)
Yes	1,207 (3.9)
History of Cardiovascular Disease (*n* = 30,574)	
No	27,520 (90.0)
Yes	3,054 (10.0)
Number of Remaining Teeth (*n* = 32,541)	
Completely edentulous (no teeth)	942 (2.9)
Partial Dentition (1–27 teeth)	19,465 (59.8)
Complete Dentition (28 teeth)	12,134 (37.3)
Functional Dentition Status (*n* = 32,541)	
No	7,908 (24.3)
Yes	24,633 (75.7)

a The group “other” includes those who identified as Hawaiian/Pacific Islander, Native American/Alaska Native/Inuit, or more than one race.

**Table 2:** Associations between the number of remaining teeth and cardiometabolic risk factors.

Variable	N	Median NRT (IQR)	*P*-Value
Age Categorical (*n* = 32,541)			**<0**.**001**^a^
18–39 years	10,539	28.0 (27.0, 28.0)^c^	
40–64 years	14,638	25.0 (20.0, 28.0)^d^	
65–74 years	4,835	21.0 (14.0, 25.0)^e^	
75–89 years	2,529	19.0 (10.0, 24.0)^f^	
Gender (*n* = 32,541)			0.523^b^
Female	17,594	26.0 (21.0, 28.0)	
Male	14,947	26.0 (21.0, 28.0)	
Race (*n* = 32,541)			**<0**.**001**^a^
White/Caucasian	7,148	25.0 (20.0, 28.0)^c^	
Black/African American	697	26.0 (20.0, 28.0)^d^	
Asian	5,760	27.0 (23.0, 28.0)^e^	
Missing	18,713	26.0 (21.0, 28.0)^e^^,^^f^	
Other	223	27.0 (22.0, 28.0)^d^^,^^e^^,^^f^	
Ethnicity (*n* = 32,541)			**<0**.**001**^a^
Hispanic/Latino	4,230	26.0 (21.8, 28.0)^c^	
Non-Hispanic/Non-Latino	10,031	25.0 (20.0, 28.0)^d^	
Missing	18,280	26.0 (21.0, 28.0)^e^	
Body Mass Index (*n* = 24,147)			**<0**.**001**^a^
Underweight (<18.5 kg/m^2^)	379	27.0 (21.0, 28.0)^c^	
Healthy weight (18.5 to <25 kg/m^2^)	6,973	26.0 (21.0, 28.0)^c^	
Overweight (25–29.99 kg/m^2^)	8,707	25.0 (20.0, 28.0)^d^	
Obese (≥30 kg/m^2^)	8,088	25.0 (20.0, 28.0)^d^	
Smoking Status (*n* = 23,617)			**<0**.**001**^b^
No	19,839	26.0 (21.0, 28.0)	
Yes	3,778	25.0 (18.0, 28.0)	
Diabetes (*n* = 31,335)			**<0**.**001**^b^
No	26,561	26.0 (22.0, 28.0)	
Yes	4,774	22.0 (15.0, 27.0)	
Hypertension (*n* = 30,636)			**<0**.**001**^b^
No	20,871	27.0 (23.0, 28.0)	
Yes	9,765	23.0 (16.0, 27.0)	
Stroke (*n* = 31,282)			**<0**.**001**^b^
No	30,075	26.0 (21.0, 28.0)	
Yes	1,207	20.0 (12.0, 26.0)	
Cardiovascular Disease (*n* = 30,574)			**<0**.**001**^b^
No	27,520	26.0 (22.0, 28.0)	
Yes	3,054	21.0 (13.0, 26.0)	

a *P*-values are based on the Kruskal–Wallis test. Bold values are significant at *P* < 0.05, adjusted for multiple comparisons using the Bonferroni method.

b *P*-values are based on the Mann–Whitney *U*-test. Bold values are significant. *P* < 0.008 was considered statistically significant after considering multiple comparisons using the Bonferroni method (0.05/6).

c–f Values with differing letters as a superscript are significantly different at *P* < 0.05 based on Bonferroni-adjusted *post-hoc* pairwise comparisons.

**Table 3:** Associations between functional dentition Status and cardiometabolic risk factors.

Variable	No FDS *n* (%)	FDS *n* (%)	*P*-value^c^
Age categorical (*n* = 32,541)			**<0** **.** **001**
18–39 years	279 (3.5)^a^	10,260 (41.7)^b^	
40–64 years	3,802 (48.1)^a^	10,836 (44.0)^b^	
65–74 years	2,352 (29.7)^a^	2,483 (10.1)^b^	
75–89 years	1,475 (18.7)^a^	1,054 (4.3)^b^	
Gender (*n* = 32,541)			0.666
Female	4,259 (53.9)	13,335 (54.1)	
Male	3,649 (46.1)	11,298 (45.9)	
Race (*n* = 32,541)			**<0** **.** **001**
White/Caucasian	1,973 (24.9)^a^	5,175 (21.0)^b^	
Black/African American	1,556 (19.7)^a^	4,204 (17.1)^b^	
Asian	131 (1.7)^a^	566 (2.3)^b^	
Missing	4,208 (53.2)^a^	14,505 (58.9)^b^	
Other	40 (0.5)^a^	183 (0.7)^b^	
Hispanic/Latino	922 (11.7)^a^	3,308 (13.4)^b^	
Non-Hispanic/Non-Latino	2,773 (35.1)^a^	7,258 (29.5)^b^	
Missing	4,213 (53.3)^a^	14,067 (57.1)^b^	
Body Mass Index, kg/m^2^ (*n* = 24,147)			**<0** **.** **001**
Underweight (< 18.5 kg/m^2^)	94 (1.5)^a^	285 (1.6)^a^	
Healthy weight (18.5 to <25 kg/m^2^)	1,615 (26.1)^a^	5,358 (29.8)^b^	
Overweight (25–29.99 kg/m^2^)	2,276 (36.8)^a^	6,431 (35.8)^a^	
Obese (≥30 kg/m^2^)	2,205 (35.6)^a^	5,883 (32.8)^b^	
Smoking Status (*n* = 23,617)			**<0** **.** **001**
No	4,354 (78.2)^a^	15,487 (85.8)^b^	
Yes	1,212 (21.8)^a^	2,566 (14.2)^b^	
Diabetes (*n* = 31,335)			**<0** **.** **001**
No	5,641 (73.8)^a^	20,920 (88.3)^b^	
Yes	2,005 (26.2)^a^	2,769 (11.7)^b^	
Hypertension (*n* = 30,636)			**<0** **.** **001**
No	3,500 (46.8)^a^	17,371 (75.0)^b^	
Yes	3,981 (53.2)^a^	5,784 (25.0)^b^	
Stroke (*n* = 31,282)			**<0** **.** **001**
No	7,038 (92.1)^a^	23,037 (97.4)^b^	
Yes	604 (7.9)^a^	603 (2.6)^b^	
Cardiovascular Disease (*n* = 30,574)			**<0** **.** **001**
No	6,018 (80.6)^a^	21,502 (93.0)^b^	
Yes	1,446 (19.4)^a^	1,608 (7.0)^b^	

FDS, functional dentition status.

a,b Proportions with different superscript letters within a row are significantly different (*P* <  0.05), based on Bonferroni-adjusted *post-hoc* pairwise comparisons of cell proportions.

c *P* values derived from the chi-square test. Bold values are statistically significant at *P* < 0.005 after accounting for multiple testing through Bonferroni correction (0.05/10).

In multivariable model 1 (Table 4), which adjusted for modifiable and non-modifiable CMRFs (sociodemographic and lifestyle characteristics), each additional remaining tooth was significantly associated with 2% lower odds of having a history of DM (OR = 0.98, 95% CI = 0.98, 0.99), 2% lower odds of having HTN (OR = 0.99, 95% CI = 0.98, 0.99), 3% lower odds of having a stroke (OR = 0.97, 95% CI = 0.96, 0.98), and 3% lower odds of having CVD (OR = 0.97, 95% CI = 0.97, 0.98). In multivariable model 2 (Table 4), which adjusted for the confounders in model 1 as well as all of the cardiometabolic diseases (DM, HTN, stroke, and CVD, where appropriate), each additional remaining tooth was significantly associated with 1% lower odds of having DM (OR = 0.9, 95% CI = 0.8, 0.99), having HTN (OR = 0.99, 95% CI = 0.98, 1.00), and 2% lower odds of having CVD (OR = 0.98, 95% CI = 0.97, 0.99); however, association between the NRT and stroke was no longer significant.

**Table 4:** Associations between the number of remaining teeth and cardiometabolic diseases^a^.

Variable	Crude model^b^		Multivariable model 1^c^		Multivariable model 2^d^	
	OR (95% CI)	*P*	OR (95% CI)	*P*	OR (95% CI)	*P*
Outcome						
History of Diabetes, Yes	0.94 (0.937, 0.944)	**<0** **.** **001**	0.98 (0.98, 0.99)	**<0** **.** **001**	0.99 (0.98, 0.99)	**<0** **.** **001**
History of Hypertension, Yes	0.92 (0.916, 0.922)	**<0** **.** **001**	0.98 (0.98, 0.99)	**<0** **.** **001**	0.99 (0.98, 1.00)	**<0** **.** **001**
History of Stroke, Yes	0.93 (0.927, 0.939)	**<0** **.** **001**	0.97 (0.96, 0.98)	**<0** **.** **001**	0.99 (0.98, 1.00)	**0** **.** **098**
History of Cardiovascular Disease, Yes	0.93 (0.926, 0.934)	**<0** **.** **001**	0.97 (0.97, 0.98)	**<0** **.** **001**	0.98 (0.97, 0.99)	**<0** **.** **001**

a Reference category includes history of diabetes (no), history of hypertension (no), history of stroke (no), and history of cardiovascular disease (no). Number of remaining teeth is the predictor variable on a continuous scale, and cardiometabolic diseases are the outcome on a dichotomous scale in each model. For the crude model, given multiple separate regression models were run for different outcomes, a Bonferroni correction of *P* ≤ 0.01 (0.05/4) was applied. Statistically significant *P* values are bolded.

b No adjustment was made. History of diabetes (*n* = 31,335), history of hypertension (*n* = 30,636), history of stroke (*n* = 31,282), and history of cardiovascular disease (*n* = 30,574).

c Adjusted for age, gender, BMI, race, ethnicity, and smoking status. History of diabetes (*n* = 19,524), history of hypertension (*n* = 19,520), history of stroke (*n* = 19,531), and history of cardiovascular disease (*n* = 19,529).

d Adjusted for all confounders in model 1 as well as self-reported history of diabetes, hypertension, stroke, and cardiovascular disease where appropriate (*n* = 19,503).

FDS was similarly significantly associated with all cardiometabolic diseases in the crude model and Model 1 (all *P* < 0.001) (Table 5). Those with a FD were 13% less likely to report a history of DM (OR = 0.77, 95% CI = 0.70, 0.84), 22% less likely to report a history of HTN (OR = 0.78, 95% CI = 0.72, 0.85), 35% less likely to report a history of stroke (OR = 0.65, 95% CI = 0.55, 0.77), and 29% less likely to report a history of CVD (OR = 0.71, 95% CI = 0.63, 0.79). In the fully adjusted model 2, the observed associations in adjusted model 1 remained significant; those with a FD were 18% less likely to report a history of DM (OR = 0.82, 95% CI = 0.75, 0.90) than those without a FD; those with a FD were 16% less likely to report a history of HTN (OR = 0.84, 95% CI = 0.77, 0.92) and 19% less likely to report a history of CVD (OR = 0.81, 95% CI = 0.71, 0.93), respectively, than those without a FD.

**Table 5:** Associations between the functional dentition Status and cardiometabolic diseases^a^.

Variable	Crude model^b^		Multivariable model 1^c^		Multivariable model 2^d^	
	OR (95% CI)	*P*	OR (95% CI)	*P*	OR (95% CI)	*P*
Outcome						
History of Diabetes, Yes	0.37 (0.35, 0.40)	**<0** **.** **001**	0.77 (0.70, 0.84)	**<0** **.** **001**	0.82 (0.75, 0.90)	**<0** **.** **001**
History of Hypertension, Yes	0.29 (0.28, 0.31)	**<0** **.** **001**	0.78 (0.72, 0.85)	**<0** **.** **001**	0.84 (0.77, 0.92)	**<0** **.** **001**
History of Stroke, Yes	0.31 (0.27, 0.34)	**<0** **.** **001**	0.65 (0.55, 0.77)	**<0** **.** **001**	0.85 (0.70, 1.05)	**0** **.** **127**
History of Cardiovascular Disease, Yes	0.31 (0.29, 0.34)	**<0** **.** **001**	0.71 (0.63, 0.79)	**<0** **.** **001**	0.81 (0.71, 0.93)	**0** **.** **002**

a Reference category includes history of diabetes (no), history of hypertension (no), history of stroke (no), and history of cardiovascular disease (no). Number of remaining teeth is the predictor variable on a continuous scale and cardiometabolic diseases are the outcome on a dichotomous scale in each model. For the crude model, given multiple separate regression models were run for different outcomes, a Bonferroni correction of *P* ≤ 0.01 (0.05/4) was applied. Statistically significant *P* values are bolded.

b No adjustment was made. History of diabetes (*n* = 31,335), history of hypertension (*n* = 30,636), history of stroke (*n* = 31,282), and history of cardiovascular disease (*n* = 30,574).

c Adjusted for age, gender, BMI, race, ethnicity, and smoking status. History of diabetes (*n* = 19,524), history of hypertension (*n* = 19,520), history of stroke (*n* = 19,531), and history of cardiovascular disease (*n* = 19,529).

d Adjusted for all confounders in model 1 as well as self-reported history of diabetes, hypertension, stroke, and cardiovascular disease where appropriate (*n* = 19,503).

There were significant interactions between NRT and age (*P_int_ *< 0.001) and BMI (*P_int_ *= 0.006) for a history of DM, and significant interactions between NRT and age (*P_int_ *< 0.001), gender (*P_int_ *= 0.03), and BMI (*P_int_ *= 0.003) for a history of HTN (Table 6). The associations were slightly stronger in patients who were younger (18–39 years old) and underweight (BMI <18.5 kg/m^2^) than in those who were 40 years and older and those with a BMI of 18.5 kg/m^2^ and higher.

**Table 6:** Associations between the number of remaining teeth and cardiometabolic diseases stratified by age, gender, and BMI^a^.

Variable	*n* ^b^	History of diabetes		History of hypertension	
		OR (95% CI)^c^	*P-int*	OR (95% CI)^c^	*P-int*
Age					
18–39 years old	5,856	0.96 (0.91, 0.99)	<0.001	0.92 (0.90, 0.95)	<0.001
40–64 years old	9,103	0.98 (0.97, 0.98)		0.97 (0.96, 0.98)	
65–74 years old	3,030	0.98 (0.97, 0.99)		0.99 (0.98, 0.99)	
75–89 years old	1,514	0.98 (0.97, 0.99)		0.99 (0.98, 1.00)	
Gender					
Female	10,591	0.98 (0.97, 0.99)	0.053	0.99 (0.98, 0.99)	0.03
Male	8,912	0.99 (0.98, 0.99)		0.99 (0.98, 0.99)	
Body mass index (kg/m^2^)					
Underweight (<18.5)	292	0.97 (0.90, 1.04)	0.006	0.95 (0.90, 0.99)	0.003
Healthy weight (18.5- 24.9)	5,579	0.98 (0.97, 0.99)		0.99 (0.98, 0.99)	
Overweight (25.0–29.99)	7,092	0.98 (0.97, 0.99)		0.98 (0.98, 0.99)	
Obese (≥30)	6,540	0.99 (0.98, 0.99)		1.00 (0.99, 1.01)	

a Reference category includes a history of diabetes (no) and, history of hypertension (no).

b n was similar across all strata for cardiometabolic diseases.

c Adjusted for age, gender, BMI, race, ethnicity, smoking status, as well as self-reported history of diabetes, hypertension, stroke, and cardiovascular disease where appropriate.

Significant interactions were observed between FDS and age (*P_int_ *< 0.001), gender (*P_int_ *= 0.031), and BMI (*P_int_ *= 0.01) for a history of DM. Also, there were significant interactions between FDS and age (*P_int_ *< 0.001), and BMI (*P_int_ *= 0.037) for a history of HTN, and a significant interaction between FDS and gender (*P_int_ *= 0.024) for a history of stroke (Table 7). Overall, the associations were stronger in patients who were younger (18–39 years old) compared to those who were 40–64 years old, 65–74 years old, and 75–89 years old. No significant interactions were found between race, ethnicity, and tobacco use and either NRT or FDS for any of the cardiometabolic diseases.

**Table 7:** Associations between the functional dentition Status and cardiometabolic diseases stratified by Age, gender, and BMI^a^.

Variable	*n* ^b^	History of diabetes		History of hypertension		History of stroke	
		OR (95% CI)^c^	*P-int*	OR (95% CI)^c^	*P-int*	OR (95% CI)^c^	*P-int*
Age							
18–39 years old	5,856	0.46 (0.22, 0.99)	<0.001	0.39 (0.23, 0.65)	<0.001	0.96 (0.05, 18.31)	0.089
40–64 years old	9,103	0.72 (0.63, 0.81)		0.70 (0.63, 0.78)		0.71 (0.52, 0.96)	
65–74 years old	3,030	0.83 (0.70, 0.98)		0.77 (0.66, 0.90)		0.97 (0.68, 1.37)	
75–89 years old	1,514	0.72 (0.57, 0.91)		0.74 (0.58, 0.93)		1.06 (0.69, 1.63)	
Gender							
Female	10,591	0.75 (0.66, 0.86)	0.031	0.88 (0.78, 0.98)	0.511	1.08 (0.81, 1.45)	0.024
Male	8,912	0.90 (0.78, 1.03)		0.80 (0.71, 0.91)		0.70 (0.53, 0.93)	
Body mass index (kg/m^2^)							
Underweight (<18.5)	292	0.34 (0.09, 1.35)	0.01	0.73 (0.28, 1.95)	0.037	0.42 (0.006, 30.34)	0.996
Healthy weight (18.5–24.9)	5,579	0.69 (0.55, 0.86)		0.82 (0.69, 0.97)		0.86 (0.57, 1.31)	
Overweight (25.0–29.99)	7,092	0.84 (0.72, 0.98)		0.79 (0.69, 0.90)		0.73 (0.52, 1.03)	
Obese (≥30)	6,540	0.88 (0.76, 1.01)		0.93 (0.81, 1.07)		0.99 (0.72, 1.38)	

a Reference category includes a history of diabetes (no) and, history of hypertension (no).

b n was similar across all strata for cardiometabolic diseases.

c Adjusted for age, gender, BMI, race, ethnicity, smoking status, as well as self-reported history of diabetes, hypertension, stroke, and cardiovascular disease where appropriate.

## Discussion

The main objective of this study was to explore associations between tooth loss and cardiometabolic diseases while controlling for CMRFs among adults seen for routine care at an urban Northeast US school of dental medicine (RSDM). The primary findings of this study were that those with more teeth and an FD were significantly less likely to have a history of DM, HTN, and CVD than those with fewer teeth and/or a lack of an FD, after adjusting for sociodemographic and lifestyle CMRFs, supporting our hypothesis. Notably, these data are the first findings to be published on a US cohort in over a decade and build upon prior research. Zhu et al. (25) found that having higher BP was associated with having fewer teeth, and Zhang et al. (26) found that a greater degree of tooth loss was associated with a higher risk of stroke. Similar to our findings, both Zhu et al. (25) and Zhang et al. (26) reported that a greater degree of tooth loss was associated with a history of DM, impaired glycemic control, and CVD. Even in the fully adjusted model, having more teeth and a FD were associated with significantly lower odds of having DM, HTN, and CVD. These findings are consistent with Helfand et al.'s (21) 2009 meta-analysis, which demonstrated a higher risk of CVD among those with fewer remaining teeth, and that of Dietrich et al. (20), who found that tooth loss was more prevalent among those with CVD. Although this and other studies defined the criteria for conditions such as CVD, HTN, and DM differently, making direct comparisons challenging, this body of research supports that those with DM, HTN, and CVD, are more likely to have poor dentition. These findings may not be generalizable to all populations as the sociodemographic profiles of different populations vary, which in turn affects their CMR.

The major strength of this study includes its large sample size, derived from a culturally and ethnically diverse patient population at RSDM, an urban northeastern US dental school clinic. Unlike other studies where dentition status was self-reported, this study measured dentition status through clinical examination by trained dental professionals. We also controlled for several potential confounding CMRFs, which are commonly assessed as part of validated tools for measuring CMR (27, 29, 30, 32). Nonetheless, we cannot completely rule out the possibility of some residual and unmeasured confounding. Furthermore, FDS (10), a clinically relevant measure of a patient's dentition status and functional chewing ability, was also analyzed in regression models.

Limitations include that this study relied on self-reported data for many of the CMRF and cardiometabolic diseases. Gender, race, and ethnicity were self-selected demographic categorical variables; however, in reality, they may not be binary and, as such, may modify CMR differently than previously proposed. Although patients were asked about their current use of tobacco, their history of tobacco use was not captured. Furthermore, we cannot completely rule out the possibility of some residual and unmeasured confounding, as biochemical data and data to analyze diet and physical activity patterns were not available for analysis as part of this study. A substantial amount of race and ethnicity data (∼55%) was missing, as patients often opted not to disclose these data. This refusal to disclose has no effect on their treatment at the dental school. We did not impute missing data for race and ethnicity, as these are sensitive and socially constructed data and not predictable from other variables. However, we assigned a category for missing data on race and ethnicity and included it in both bivariate and regression analyses. Additionally, data were missing for∼5%–30% of the other clinical and lifestyle variables. Given the extent of missing data for some confounding variables, we excluded cases with missing data from the regression analyses where applicable, which may limit the generalizability. However, the fully adjusted model still had a large sample size of ∼6,700 with complete data. The handling of missing data and the reliance on self-reported clinical conditions may have affected the internal validity and generalizability of our findings. Despite being a racially and ethnically diverse sample representative of our patient population, the findings may not be universally generalizable elsewhere (34). Finally, the study design was cross-sectional; thus, causality cannot be inferred from the findings.

### Implications for practice and future research

While prior research has laid the foundation for exploring the associations between dentition status and cardiometabolic health and disease (22–26), this was the first published study to be conducted in a US population in over a decade. Furthermore, this study was unique in that dentition status was measured by clinical examination, rather than self-report, and functional dentition was analyzed as both a dichotomous variable and a continuous scale, based on the number of remaining teeth. Thus, these findings contribute to a more nuanced understanding of the associations between dentition status and cardiometabolic diseases, as well as their common risk factors. As such, tooth loss can serve as a marker for increased risk of cardiometabolic diseases. Enhanced knowledge and awareness of these associations can lead to improved CMR screening practices in an interprofessional setting, such as the dental clinic. Research suggests that people are more likely to visit their dentist than their physician, making this an ideal setting to optimize CMR screening (35). A greater understanding of these associations among clinicians can also lead to an increased willingness to initiate discussions and engage in patient education, which, in turn, can enhance patient awareness and willingness to undergo screening. CMR screening may enable earlier referrals for individuals at higher risk, which could then lead to interventions targeted at prevention and treatment, as appropriate (4). These findings also support the Healthy Smiles, Healthy Hearts initiative of the American Heart Association, in collaboration with Delta Dental, to educate oral health professionals and engage them in CVD risk/BP screening, with referrals to primary care physicians, as appropriate (36, 37).

Further research, particularly with longitudinal designs, is warranted to assess the effects of tooth loss over time on CVD progression, thereby advancing research in this area. Future studies should include large and diverse samples with consistent measurement and reporting of additional biomarkers, including relevant laboratory and diagnostic measures associated with CMR, such as lipid profiles, hemoglobin A1C, and inflammatory markers. The inclusion of behavioral data, such as measurements of dietary and physical activity using validated assessment tools, would further strengthen future research related to CMR. Prospective studies would enable the exploration of the effects of progressive changes in dental status on cardiometabolic health and disease risk.

## Conclusion

In this urban northeast U.S. sample of adults, those with more teeth and a functional dentition were less likely to have cardiometabolic diseases even after controlling for sociodemographic and lifestyle CMRFs. Future research would benefit from the standardization and consistency of measures to facilitate comparisons of findings. Enhanced knowledge and awareness of these associations can lead to improved cardiometabolic risk screening in interprofessional settings.

## Data Availability

The datasets used and/or analyzed during the current study can be made available from the corresponding author upon reasonable request if approved by the IRB. Requests to access the datasets should be directed to renazelig@gmail.com.
